# Supplementary material for: Inhibitory Effect on Nitric Oxide Release in LPS-Stimulated Macrophages and Free Radical Scavenging Activity of Croton Linearis Jacq. Leaves
Source: Antioxidants (Basel). 2022 Sep 27;11(10):1915. doi: 10.3390/antiox11101915 (PMC9598791; doi:10.3390/antiox11101915)
Supplement: Supplementary file 1 [file antioxidants-11-01915-s001.zip › antioxidants-1901620-supplementary.pdf]

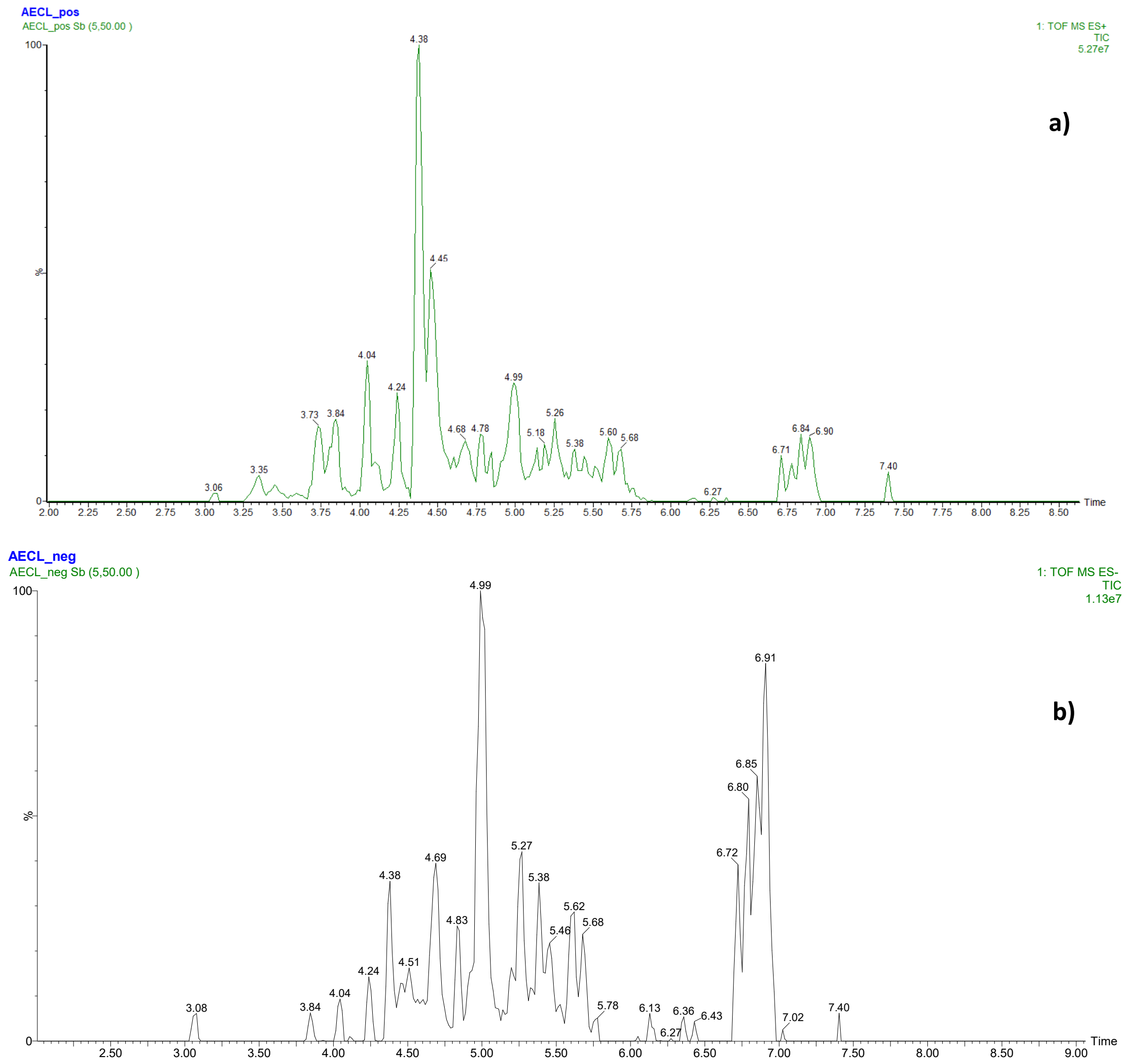

Figure S1. UPLC-QTOF-MS chromatogram of AE fraction from *Croton linearis* Jacq leaves: **a)** positive ion mode, **b)** negative ion mode.

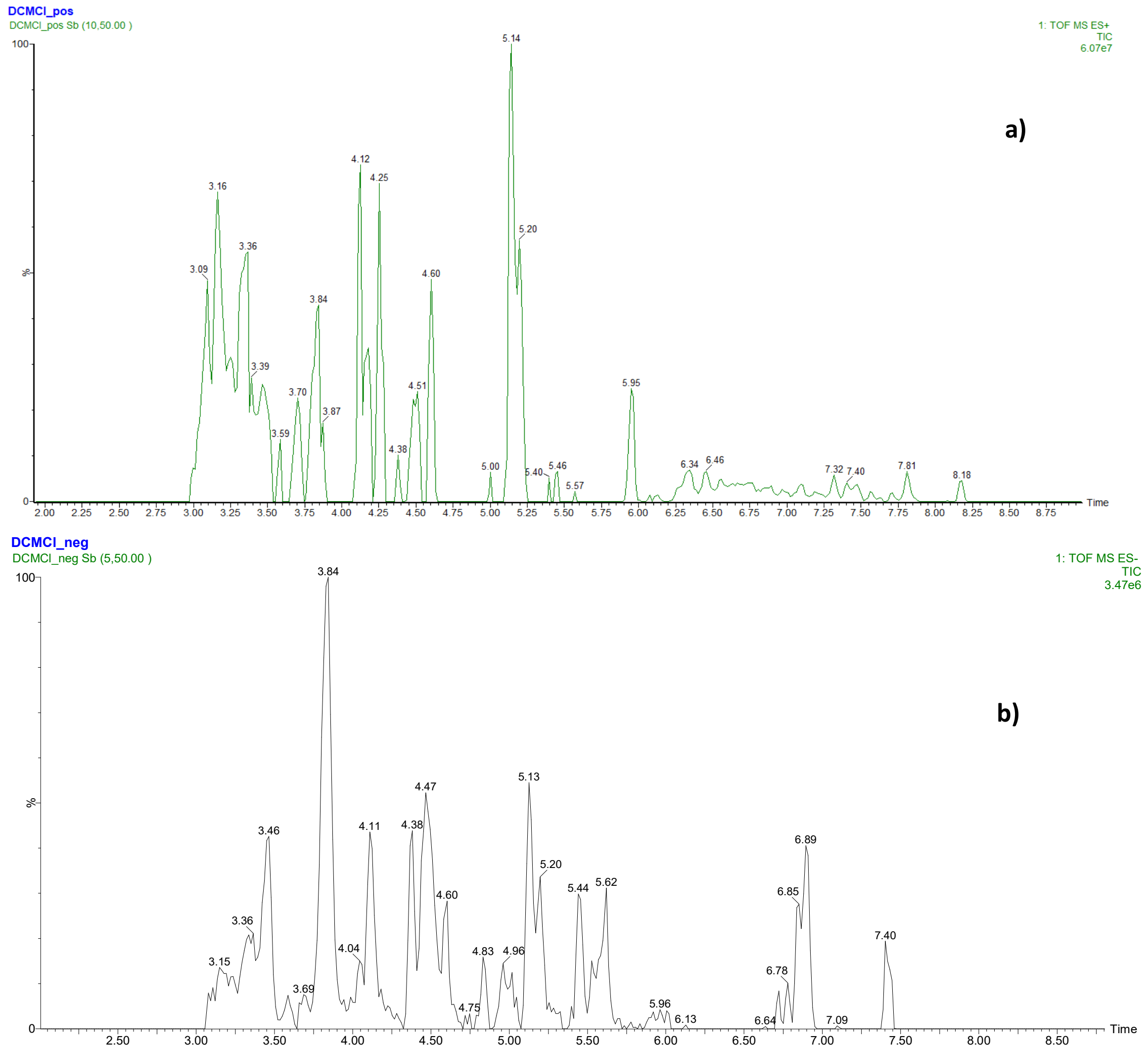

Figure S2. UPLC-QTOF-MS chromatogram of DCM fraction from *Croton linearis* Jacq leaves: **a)** positive ion mode, **b)** negative ion mode.
